# Homocysteine, folate, hs-C-reactive protein, tumor necrosis factor alpha and inflammatory proteins: are these biomarkers related to nutritional status and cardiovascular risk in childhood-onset systemic lupus erythematosus?

**DOI:** 10.1186/s12969-017-0220-y

**Published:** 2018-01-09

**Authors:** Roberta Garcia Salomão, Luciana Martins de Carvalho, Clarice Izumi, Érika Silva Czernisz, José César Rosa, Sonir Roberto Rauber Antonini, Ana Carolina Bueno, Maria Olímpia Ribeiro do Vale Almada, Carolina de Almeida Coelho-Landell, Alceu Afonso Jordão, Virgínia Paes Leme Ferriani, Jacqueline Pontes Monteiro

**Affiliations:** 1 Department of Pediatrics, Ribeirão Preto Medical School, University of São Paulo, Av. Bandeirantes, 3900, 14049-900 Ribeirão Preto, São Paulo, Brasil; 2 Protein Chemistry Center, Ribeirão Preto Medical School, University of São Paulo, Ribeirão Preto, Brasil; 3 Department of Medical Clinical, Ribeirão Preto Medical School, University of São Paulo, Ribeirão Preto, Brasil

**Keywords:** Proteomic, Nutritional status, Homocysteine, Folic acid, Tumor necrosis factor alpha, Childhood onset systemic lupus Erythematosus

## Abstract

**Background:**

Childhood-onset systemic lupus erythematosus (c-SLE) is a chronic autoimmune disease which increases cardiovascular risk factors (CRF) such as elevated homocysteine, TNF-α, and hs-C reactive protein.

**Methods:**

We evaluated BMI, waist circumference (WC), 24-h recalls, SLEDAI-2 K, SLICC/ACR-DI, serum levels of homocysteine, folate, TNF-α, hs-C reactive protein, lipid profile, proteomic data, and duration of corticosteroid therapy in 19 c-SLE and 38 healthy volunteers. Physiological and anthropometric variables of c-SLE and healthy controls were compared by ANCOVA. k-cluster was used to separate c-SLE into two different groups with the best and the worst metabolic profile according to previous analysis showing some metabolites that were statistically different from controls, such as homocysteine, TNF-α**,** hs-CRP and folate levels. These two clusters were again compared with the control group regarding nutritional parameters, lipid profile and also proteomic data.

**Results:**

Individuals with c-SLE presented higher BMI, WC, homocysteine, triglycerides, TNF-α**,** hs-CRP and lower folate levels when compared to controls. We found 10 proteins whose relative abundances were statistically different between control group and lupus clusters with the best (LCBMP) and the worst metabolic profile (LCWMP). A significant positive correlation was found between TNF-α and triglycerides and between hs-CRP and duration of corticosteroid therapy.

**Conclusion:**

Cardiovascular disease (CVD) risk parameters were worse in c-SLE. A less protective CVD proteomic profile was found in LCWMP.

## Background

Cardiovascular disease (CVD) is a leading cause of morbidity and mortality in adult SLE and has been associated with chronic inflammation [1]. Although there has been a significant improvement in all-cause mortality rates in adult SLE patients over time, the mortality secondary to atherosclerosis and cardiovascular disease has not significantly changed [2]. One study reporting the long-term burden of CVD in c-SLE, demonstrated that patients with onset of SLE during childhood had a similar incidence of myocardial infarction (MI) compared to subjects with adult-onset disease (7.9% versus 4.5%, respectively; *p* = 0.23), and also had the first MI earlier in life (mean of 32 years old) [3].

The incidence of acute myocardial infarction and risk of premature atherosclerosis is approximately 50 times greater in women with SLE compared to healthy controls. This association is the result of the complex interaction between classic SLE risk factors, chronic inflammation, elevated concentrations of homocysteine, the production of autoantibodies, and SLE treatment [4].

Elevated homocysteine (Hcy) levels are associated with increased risk for CVD and can indicate micronutrient insufficiencies especially cobalamin and/or folate which are required cofactors for Hcy metabolism through the remethylation pathway [5]. Inflammatory biomarkers such as high-sensitivity C-reactive protein (hs-CRP) [6] and tumor necrosis factor – α (TNF – α) [7] have been consistently associated with the presence of CVD in multiple studies in different populations including individuals with SLE.

The status of the inflammatory system has been analyzed in multiomic studies which included associations of gene variants (genomics), mRNA (transcriptomics), protein (proteomics), and metabolites (metabolomics) [8]. Until now, only one study analyzed selected plasma proteins in c-SLE [9]. The present study describes and compares anthropometric measurements, food intake, levels of folate, plasma proteins, and the cardiovascular risk factors homocysteine, TNF-α, hs-C reactive protein in c-SLE and healthy controls. These cardiovascular risk factors were correlated with nutritional status, lipid profile, and duration of corticosteroid therapy in c-SLE. We tested the hypothesis that nutritional status, lipid and proteomic profile, homocysteine, folate, hs-CRP and TNF-α levels would be consistent with CRF in c-SLE compared to the levels in healthy controls.

## Methods

### Study design and subjects

We performed a cross-sectional, cumulative incidence case control study (i.e., traditional case-control) in SLE female adolescents, between 10 to 18 years old attending a tertiary Pediatric Rheumatology Clinic with at least four criteria for Lupus diagnosis according to *American College of Rheumatology* (ACR) [10]. All female patients followed up in this Clinic were recruited to participate. Exclusion criteria were individuals with infection, diabetes, smokers, or who were pregnant.

The control group were non-smokers and non-pregnant healthy adolescents recruited from Primary Health Care Outpatient Clinic in the northeastern region of the state of São Paulo in Brazil, matched for age and pubertal stage. Individuals in the case group were obtained by convenience sampling and the sample size was based on Schlesselman (1982) defined by alpha 0.05 and beta 10% [11]. Individuals recruited for the study read and signed the Consent Form after detailed explanation about the study protocol. The study was approved by the Ethics Committee at the Clinical Hospital of the University of São Paulo in Ribeirão Preto – Brazil (process number 9946/2010) and the parents of all adolescents gave written informed consent for their participation.

### Nutritional assessment

The anthropometric evaluation involved measurements of weight, height, and waist circumference. Weight and height were evaluated according to Heymsfield and Tighe method to generate body mass index (BMI) [12] by a trained dietitian. Waist circumference (WC) was measured according to published methods [13].

Food intake was assessed with three 24 h-recalls (24 h–recall) administered to each participant. Two 24 h–recalls were done by telephone in the week prior data collection and one in the collection day [14]. In order to minimize bias inherent to the 24 h–recall instrument, the ones collected through telephone were double-checked in data collection day by a trained dietitian. A book of photos of food amounts (small, medium and large) was used [15] to avoid under and overestimation of food portion size. DietWin version 2011**®** was used to calculate intakes of energy, folate, and cyanocobalamin. Thereafter, a Multiple Source Method that used regression logistic to exclude intra- and inter-variability in food intake and calculate the average usual intake.

### Clinical evaluation

The clinical data were obtained from the patients’ medical records. A board certificated pediatric rheumatologist assessed blood pressure, pubertal status following the Tanner criteria [16]. The Systemic Lupus Erythematosus Disease Activity Index e (SLEDAI 2 k) [17] and Systemic Lupus International Collaborating Clinics/American College of Rheumatology Damage Index (SLICC/ACR DI) [18] were used to evaluate the disease activity and cumulative damage, respectively. In addition, the pediatric rheumatologist calculated prednisone dose, in milligrams per kilogram per day, for use during the 6 month prior to the study.

### Biochemical parameters

Homocysteine, folate, TNF-α and hs-CRP levels were measured according to a chemiluminescent technique (IMMULITE 1000**®)**. Cholesterol levels were determined by enzymatic method and high-density lipoprotein (HDL) and low-density lipoprotein (LDL) by colorimetric method (Wiener Lab®).

### Shotgun proteomics – Isobaric tag for relative and absolute quantitation (iTRAQ)

Delipidation of plasma was performed according to Fu et al. (2005) [19]. Albumin and IgG were depleted by immunodepletion with Proteopep Immunoaffinity Albumin and IgG depletion Kit (Sigma®). The samples were reduced and alkylated, digested with trypsin, and labeled with iTRAQ 8-plex reagents (AB Sciex®) according to manufacturer’s instructions. After labeling, all samples were combined in a single tube forming a single pool. This pool was mixed and fractionated into 40 fractions by strong cation exchange chromatography (PolyLC – Polysulfoethyl A). Eluted peptides were sprayed through a 10 μm emitter tip into an ESI-Q-TOF interfaced with a NanoAcquity ultra-HPLC (Waters) [20].

Mass spectrometer (MS/MS) spectra were extracted with and without de-convolution using Thermo Scientific Xtract® software and searched against the RefSeq 40 protein database using the Mascot Distiller™ (Matrix Science) through Proteome Discover software (v1.3, Thermo Scientific). Peptide identifications from Mascot searches were filtered within the Proteome Discover to identify peptides with ≥95% confidence [i.e., false discovery rate (FDR) <5%] [20].

The subset of proteins identified were quantitatively analyzed in samples of all participants using Scaffold Q+ (Proteome Software Inc., Portland, OR) based on centroid reporter ion intensity. The mass spectrometer generates peaks based on mass/charge (m/z) of each peptide present in the sample. The area of the peak in the MS chromatogram provides a measure of the relative abundance of the corresponding peptide in the sample. The areas under these peaks are calculated and normalized and their ratios are used as measure of the relative abundance of the peptides in different samples. The relative abundance was based on the ratio between lupus groups and control, assuming that control group abundance would be equal 1. A variation of greater than 30% abundance of metabolites/proteins between groups or clusters was considered biologically relevant.

### Statistical analysis

The softwares SPSS® (v.20.0), Dietwin 2011®, Mascot Distiller™ (Proteome software) and Scaffold Q+ (Proteome Software Inc., Portland, OR) were used for the analyses. Variables in c-SLE and control groups were compared through covariance analysis (ANCOVA), adjusting for age, BMI, folate and cyanocobalamin intake, when applicable. Spearman test was used to correlate some variables. ANCOVA and Spearman tests were also adjusted for Bonferroni. The significance level of *p* < 0.05 was considered statistically significant for comparisons before Bonferroni test. For multiple comparisons analysis the considered significance levels were *p* = 0.005.

Abundances found in iTRAQ analyses were compared with ANOVA and post hoc Bonferroni. k-cluster analysis was used to assess the presence of different physiological groups. Only k = 2 separated c-SLE yielding the best and the worst metabolic profile. This determination was based on previous analysis showing some metabolites including homocysteine, TNF-α**,** hs-CRP, and folate serum levels that were statistically different in SLE patients from controls. The best metabolic profile (LCBMP) was considered as folate serum levels within the normal range and homocysteine, TNF-α, and hsCRP under normal ranges. The worst metabolic profile (LCWMP) was considered when folate serum levels were low and homocysteine, TNF-α, and hsCRP were above maximum range values. Thereafter, these two clusters or groups were compared with control group. An internal control group with 1/3 of the protein amount of each cluster was created to evaluate data consistency.

## Results

Twenty c-SLE subjects who met the inclusion and exclusion criteria were recruited and nineteen agreed to participate completing all follow-up procedures in the study. Forty-five healthy controls who met inclusion and exclusion criteria were recruited and thirty-eight agreed to participate in the study.

c-SLE and control groups were matched for age (c-SLE 15.7 ± 1.95 years old; Control 14.9 ± 2.5 years old, *p* = 0.117) and pubertal status (breast *p* = 0.158 and pubic hair *p* = 0.578). The age at diagnosis in lupus group was 10.8 ± 2.7 years old and mean disease duration until data collection day was 6.3 ± 2.6 years old. The median SLEDAI 2 k in c-SLE participants was 4 (range 0–29); 16/19 (84.2%) had active disease, with 4 having important activity (> 12), 3 with a moderate activity (5–12), 9 with mild activity (2–4), and 3 were classified as not having an active disease (equal to zero) [21]. The median SLICC/ACR-DI score was 0 (range 0–9) and six patients had a score of one or higher. The mean duration of steroid use was 5 years. Fifteen out of nineteen subjects used prednisone in the last 6 months prior data collection (0.36 ± 0.32 mg/kg). Systolic and diastolic blood pressure levels were not different between lupus and controls (systolic: 105.51 ± 2.26 versus 102.80 ± 1.61, *p* = 0.354; diastolic: 68.5 ± 2.19 versus 69.00 ± 1.56, *p* = 0.860).

BMI, waist circumference, Hcy, TNF- α, hs-CRP, and triglycerides levels were higher in participants with c-SLE, while serum folate was significantly lower (Table 1). No significant differences in energy (lupus 1566 ± 76 Kcal/day vs controls 1594 ± 52 Kcal/day, *p* = 0.766) or folate intake (lupus 83.91 ± 9.64 μg/day vs controls 77.30 ± 6.59 μg/day, *p* = 0.588) were found. Vitamin B12 intake was significantly higher in c-SLE participants compared to controls (lupus 3.14 ± 0.17 μg/day vs controls 2.65 ± 1.8 μg/day, *p* = 0.029). Anthropometric and biochemical parameters were also tested between control group, LCBMP, and LCWMP (Table 2) and as expected, all parameters related to CVD were statistically higher in lupus clusters compared to controls, while folate level was lower.

**Table 1:** Anthropometric and biochemical parameters in pediatric childhood-onset SLE subjects and in healthy controls

Variables	SLE Group (*n* = 19)	Control Group (*n* = 38)	*p*
BMI (kg/m^2^)^a^	24.6 (18.45–40.0)	20.83 (15.96–31.24)	0.001**
WC (cm)^a^	79.66 ± 8.94	72.25 ± 7.88	0.001**
Folate (ng/L)^c^	12.16 (5.8–19.25)	13.91 (10.4–26.2)	0.033*
Homocysteine (mmol/L)^b^	8.62 ± 2.36	7.29 ± 1.80	0.019*
Total Cholesterol (mg/dL)^c^	160.73 ± 42.76	152.75 ± 31.57	0.122
HDL (mg/dL)^c^	37.58 (27.0–61.0)	44.14 (25.0–99.0)	0.088
LDL (mg/dL)^c^	97.26 ± 26.16	89.75 ± 27.18	0.320
Triglycerides (mg/dL)^c^	152.42 (31.0–725.0)	88.16 (25.0–226.0)	0.003**
TNF - α (pg/mL)^c^	8.65 ± 2.55	7.04 ± 2.39	0.002**
hs-CRP (mg/L)^c^	4.41 (0.3–17.0)	0.84 (0.3–4.6)	0.000**

*p < 0.05; post hoc Bonferroni **p < 0.005

Values expressed as mean ± standard deviation. The difference between means was calculated by ANCOVA adjusted by: age^a^; age, BMI and cyanocobalamin intake^b^; age and BMI^c^

BMI = Body Mass Index, *WC* Waist Circumference, *HDL* High Density Lipoprotein, *LDL* = Low Density Lipoprotein, *TNF – α* Tumor Necrosis Factor – α, *Hs-CRP* high sensitive C- reactive protein

**Table 2:** Anthropometric and biochemical parameters in control group, LCBMP and LCWMP

Variables	Control group (n = 38)	LCBMP (*n* = 17)	LCWMP (*n* = 2)
BMI (kg/m^2^)^¶^	20.83 (15.96–31.24)^A*^	24.97 (20.35–40.0) ^A*^	21.51 (18.45–24.58)
WC (cm)^¶^	72.34 ± 7.97 ^A*^	80.21 ± 9.08 ^A*^	75 ± 8.48
Homocysteine (mmol/L) ^¥^	7.27 ± 1.82 ^A,B^	8.59 ± 2.38 ^A^	8.85 ± 3.04 ^B^
Folate (ng/L)^§^	13.94 (10.4–26.2) ^B*^	12.37 (5.8–19.25)	10.3 (9.4–11.2) ^B*^
Cholesterol (mg/dL)^§^	152.28 ± 31.90	157.23 ± 42.34	190.5 ± 47.37
HDL (mg/dL)^§^	44.26 (25.0–99.0)	37.93 (27.0–61.0)	35.0 (32.0–38.0)
LDL(mg/dL)^§^	88.93 ± 27.23	95.53 ± 27.49	108.5 ± 14.84
Triglycerides (mg/dL)^§^	88.65 (25.0–226.0) ^A*, B^	142.82 (31.0–725.0)^A*^	234.0 (105.0–363.0)^B^
TNF-α (pg/mL)^§^	7.08 ± 2.41^A, B*^	8.22 ± 2.14^A,C^	12.32 ± 3.71^B*,C^
hs-CRP (mg/L)^§^	0.86 (0.30–4.60) ^A*, B*^	2.97 (0.30–7.80) ^A*,C*^	16.65 (16.30–17) ^B*,C*^

Equal letter means *p* < 0.05; post hoc Bonferroni *p < 0.005

The difference between groups was calculated by ANCOVA adjusted by: age^**¶**^; age, BMI and cyanocobalamin intake^¥^; age and BMI^**§**^

A = ANCOVA between control group and LCBMP

B = ANCOVA between control group and LCWMP

C = ANCOVA between LCBMP and LCWMP

iTRAQ results identified 86 proteins in our total sample (Control, LCBMP, LCWMP) but after correcting for lysozyme and internal controls using Scaffold software, the abundance of only 10 proteins were statistically different between the three groups. These 10 proteins were involved in inflammatory processes and were more abundant in the c-SLE group (LCBMP) with the worse metabolic parameters (Table 3).

**Table 3:** Proteins identified by iTRAQ in control group, LCWMP and LCBMP

	Relative Abundance			p ANOVA Test (post hoc Bonferroni)	Function
Identified Proteins	Control	LCWMP	LCBMP		
Apolipoprotein A-I	1	0.8	2.1	< 0.0001*	Anti-inflamtatory protein; reverse cholesterol transport
α-2-macroglobulin	1	0.8	1.3	< 0.0001*	Anti-inflamtatory protein; Protease inhibition
α −1-antitripsin	1	0.7	1.5	0.0084*	Anti-inflamtatory protein; Protease inhibition
Apolipoprotein E	1	0.9	2.2	0.0086*	Anti-inflamtatory protein; Fat-soluble vitamins and cholesterol transport
Ceruloplasmin	1	0.9	1.6	0.00058*	Anti-inflamtatory protein; Copper and iron transport
Complement C3	1	0.8	1.3	0.0014*	Anti-inflamtatory protein; Complement system activation
Fibrinogen-α chain	1	0.7	0.9	0.0047*	Pro-inflamtatory protein;Coagulation and fibrinolysis
Haptoglobin	1	1.3	2.5	< 0.0001*	Anti-inflamtatory protein; Iron metabolin - antioxidant
Hemopexina	1	0.9	1.7	0.0010*	Anti-inflamtatory protein; Iron metabolin - antioxidant
Serum transferrin	1	0.8	1.7	< 0.0001*	Anti-inflamtatory protein; Iron metabolin - antioxidant

*p < 0.005

*LCWMP* Lupus Cluster with the Best Metabolic Profile, *LCBMP* Lupus Cluster with the Worst Metabolic Profile

Abundances were compared between three groups

by ANOVA, post hoc Bonferroni

Spearman test showed no association between CVD risk factors and nutritional status parameters (data not shown). TNF-α was positively correlated with triglycerides in lupus group (*r* = 0.519, *p* = 0.023). The levels of cardiovascular parameters total cholesterol (*r* = 0.575, *p* = 0.025) and triglycerides (*r* = 0.707, *p* = 0.003) were correlated with dosage of corticosteroid even adjusted by Bonferroni, hs-CRP was positively correlated with duration of corticosteroid therapy (*r* = 0.626, *p* = 0.009).

## Discussion

We confirmed the hypothesis that childhood-SLE have worse nutritional status based on lipid and proteomic profiles, homocysteine, folate, hs-CRP, and TNF-α levels when compared to healthy controls. In the present study, SLE patients showed higher BMI and WC compared to controls and with increased levels of cardiovascular risk factors (homocysteine, TNF- α, hs-CRP, and folate levels). High BMI values have been associated with increased CVD risk factors including hs-CRP in lupus, which contributes to an increase in morbidity and mortality in these patients [22, 23]. Cross sectional studies in children suggest that increased BMI is associated carotid intima and media thickness, and left ventricular mass in early or mid-adulthood [24]. Truncal obesity is a risk factor for coronary heart disease, which is frequently seen in SLE and is associated with the development of atheroma and metabolic syndrome [25]. Waist circumference (WC) is the best overall predictor of abdominal visceral obesity [24]. High waist circumference values were independent predictors of increased carotid intima media thickness and metabolic syndrome in adults with lupus (Rizk 2012).

The childhood SLE patients in this population had higher homocysteine and lower folate levels when compared to controls, which corroborated results of other studies [26]. Hyperhomocysteinemia has been identified as a risk factor for atherothrombotic events and has been related to impaired 5,10-mehtylenetetrahydrofolate reductase activity and deficiency of folate in patients with SLE, including children and adolescents [21, 26–29].

Dyslipidemia may be present in over half of the adult patients with lupus and CVD [23] and caused by multiple factors. Cytokine, autoantibodies, medications, dietary intake, renal disease, physical activity, and genetic factors are all likely important contributors [2]. Individuals at risk of CVD characteristically have higher levels of triglycerides and very-low-density lipoprotein cholesterol but lower levels of HDL [2, 22]. Triglycerides levels were higher in these childhood SLE patients compared to controls and were positively correlated with TNF-α, a well-known risk factor for CVD. In addition, total cholesterol and triglycerides were correlated with dosage of corticosteroid.

The autoimmunity and the inflammatory process of SLE are directly related to changes in lipid profile and metabolism of lipoproteins. Regardless of disease activity, SLE by itself and corticosteroid use promote pro-atherogenic lipoprotein profiles [22]. Some authors found a positive association between corticosteroid and serum triglycerides in adult-onset SLE [30], same results were found in our study.

The present study found a significant positive correlation between TNF-α and triglycerides. High cytokine levels such as TNFα (a non-specific pro-inflammatory factor may play a role in the dysregulation of lipid levels in pediatric SLE [7, 31]. TNF-α acts as mediator to inhibit lipoprotein lipase resulting in elevated levels of triglyceride, including those individuals with SLE [7]. Another inflammatory marker correlated with CRF is hs-CRP [32]. Some studies have found higher levels of hs-CRP in pediatric SLE patients compared with healthy controls [33]. High hs-CRP levels were correlated with functional and morphological alterations in the cardiovascular system [33, 34]. To our knowledge no study has found correlations of the use and duration of corticosteroid in childhood-onset systemic lupus erythematosus with hc-CRP. The positive correlation between hs-CRP level with duration of corticosteroid therapy found in our study is controversial [35].

Although folate consumption was not different between groups and not statistically correlated with homocysteine, folic acid serum levels were significantly lower in c-SLE subjects, which has not been previously reported in the literature. Lower folate serum levels were found in adult SLE patients and can be associated with hyperhomocysteinemia [26].

Individuals in the LCWMP group had lower levels of anti-inflammatory proteins, apolipoprotein A-I, apolipoprotein E, alpha-2-macroglobulin, alpha-1-antitrypsin, ceruloplasmin, complement C3, hemopexin and serum transferrin compared with healthy control group and with LCBMP group. Apolipoprotein A-I (apo A-I) acts in cholesterol homeostasis and inhibits pro-inflammatory cytokines decreasing risk of cardiovascular disease [9]. Similar results were found by others who described lower plasma abundance of apo A-I in pediatric patients with SLE when compared to healthy controls [9]. An pro-atherogenic profile characterized by decreased plasma levels of apo A-I was previously observed in adolescents with SLE [36]. The decreased abundance of apolipoprotein A I, apolipoprotein E, α-2-macroglobulin, α-1-antitripsin, ceruloplasmin, complement C3, serotransferrin, haptoglobin, hemopexin and serum transferrin was considered a CRF [9, 37–43]. Our study is the first one to describe the decreased abundance of these 10 proteins as potential early biomarkers of CRF in childhood-onset systemic lupus erythematosus.

The present study has some limitations. The cross-sectional design makes it difficult to establish a cause-and-effect relationship between CRF and nutritional status, lipid profiles, and inflammatory proteins. In addition, the sample size was small. The convenience sampling method often cannot be extrapolated to give population results and may be prone to volunteer bias. Hence, the conclusions stated in the study were limited to this specific group and cannot be extrapolated even though all participants shared a similar built environment. The proteomic method used here could not evaluate each sample individually due to cost considerations. However, the technique of grouping samples as done in the present study has been used in several published reports [44, 45]. Given these limitations, the present study is the first to identify and characterize distinct metabolic groups of cSLE patients, one of which had a proteomic profile consistent with increased risk of CVD when compared to healthy controls.

## Conclusion

The present study was the first one to confirm the hypothesis that children and adolescents with childhood-onset systemic lupus erythematosus have a less healthy nutritional status, lipid and proteomic profile, homocysteine, folate, hs-CRP and TNF-α levels when compared to healthy controls.

Children with chronic rheumatologic diseases are exposed to a vast array of pro-atherogenic insults, but the prevalence and natural history of accelerated atherosclerosis remains poorly defined. Cardiovascular disease is increasingly found in adult SLE patients which leads to significant increases in morbidity and mortality. Identifying key risk factors, developing disease-specific stratification algorithms, and implementing medical and nutritional interventions may help develop strategies to prevent or at least delay atherosclerosis in SLE patients. Multidisciplinary collaboration with pediatric rheumatologists, nutritionists, and preventive cardiologists may help optimize care in this vulnerable pediatric population. These unique results deserve further investigations to better elucidate the whole of these biomarkers in the context of nutrition and systems biology in SLE pediatric patients.

## Abbreviations

ACR: American college of rheumatology
apo A-I: Apolipoprotein A-I
apo E: Apolipoprotein E
BMI: Body mass index
CRF: Cardiovascular risk factor
c-SLE: Childhood-onset systemic lupus erytematosus
CVD: Cardiovascular disease
FDR: False discovery range
Hcy: Homocysteine
HDL: High-density lipoprotein
hs-CRP: High sensitive – C reactive protein
iTRAQ: Isobaric tag for relative and absolute quantitation
LCBMP: Lupus cluster best metabolic profile
LCWMP: Lupus cluster worst metabolic profile
LDL: Low-density lipoprotein
MI: Myocardial infarction
SLEDAI-2 K: Systemic lupus erythematosus disease activity index - 2000
SLICC/ACR-ID: The systemic lupus international collaborating clinics/american college of rheumatology damage index
TNF- α: Tumor necrosis factor – α
WC: Waist Circumferece
